# Steady-State Motion Visual Evoked Potentials Produced by Oscillating Newton's Rings: Implications for Brain-Computer Interfaces

**DOI:** 10.1371/journal.pone.0039707

**Published:** 2012-06-19

**Authors:** Jun Xie, Guanghua Xu, Jing Wang, Feng Zhang, Yizhuo Zhang

**Affiliations:** 1 School of Mechanical Engineering, Xi'an Jiaotong University, Xi'an, Shaanxi, People's Republic of China; 2 State Key Laboratory for Manufacturing Systems Engineering, Xi'an Jiaotong University, Xi'an, Shaanxi, People's Republic of China; Georgia State University, United States of America

## Abstract

In this study, we utilize a special visual stimulation protocol, called motion reversal, to present a novel steady-state motion visual evoked potential (SSMVEP)-based BCI paradigm that relied on human perception of motions oscillated in two opposite directions. Four Newton's rings with the oscillating expansion and contraction motions served as visual stimulators to elicit subjects' SSMVEPs. And four motion reversal frequencies of 8.1, 9.8, 12.25 and 14 Hz were tested. According to Canonical Correlation Analysis (CCA), the offline accuracy and ITR (mean ± standard deviation) over six healthy subjects were 86.56±9.63% and 15.93±3.83 bits/min, respectively. All subjects except one exceeded the level of 80% mean accuracy. Circular Hotelling's T-Squared test (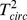) also demonstrated that most subjects exhibited significantly strong stimulus-locked SSMVEP responses. The results of declining exponential fittings exhibited low-adaptation characteristics over the 100-s stimulation sequences in most experimental conditions. Taken together, these results suggest that the proposed paradigm can provide comparable performance with low-adaptation characteristic and less visual discomfort for BCI applications.

## Introduction

Brain-computer interface (BCI) is a communication technology that bypasses the human's normal output pathways of muscle and peripheral nervous but provides a direct connection between human brain and external devices such as computers, wheelchairs [1] and robots [2]. It mostly relies on the conscious modulation of noninvasively scalp electroencephalography (EEG) to either external stimuli or internal motorsensory tasks. They correspond to exogenous and endogenous BCI applications, respectively. Over the past decades, there have been increasing research interests in both exogenous and endogenous BCIs such as the ones that are based on P300, or the ones that are based on event-related desynchronization (ERD) and event-related synchronization (ERS). Compared with the above BCI applications, one of the most popular exogenous BCIs, i.e. the steady-state visual evoked potential (SSVEP)-based BCI [3], usually has the advantages of high information transfer rate (ITR), high tolerance to artifacts and the robust performance across users. However, in the traditional SSVEP-BCI field, most researches are based on flickering or contrast-change related paradigms. There are few reports on human perception of motion [4] and its potential use in the design of steady-state BCIs.

Similar to the perception of light and contrast, motion perception is also one of the fundamental tasks of human visual system and is critical for our interactions in a dynamic environment [4]. It is typically characterized as the projection of optic flow onto the retinal surface with varying or constant depth when an object moves into a subject's environment. Expansion and contraction motions are the typical radial motions that are experienced as the relative distance change along a subject's visual axis and seem to produce higher response, rather than translation and rotation motions that are perceived perpendicular to the visual axis with no depth variation. All the direction-specific motions activate the homologous visual cortex regions along the dorsal stream and the largest neural activities often appear in areas V1 and MT/V5 [5]–[7]. Recent studies [8], [9] have utilized the motion-onset visual evoked potential (VEP), i.e. one kind of transient motion-related VEPs (mVEPs) elicited by translation motion, to construct BCI systems that are similar to P300 spellers, and have achieved promising results. Nevertheless, the perception of direction-specific motion is susceptible to adaptation, which would result in response decline and visual fatigue. In order to eliminate such effect, a sophisticated experimental design is usually necessitated to prevent successive presentation of motions in a single direction. It would bring extra difficulties in the motion-related BCI design, especially for transient mVEP-based BCIs. Our study, for the first time, utilize a special visual stimulation of non- direction-specific motion reversals to elicit the steady-state motion visual evoked potentials (SSMVEPs) for BCI application, which would reduce adaptation effect and overcome the problem of visual fatigue caused by uncomfortable light twinkling and contrast changes, especially in the low frequency range [10], [11].

## Materials and Methods

### 1 Ethics Statement

Subjects were studied after giving informed written consent in accordance with a protocol approved by the institutional review board of Xi'an Jiaotong University.

### 2 Stimulation Design

The visual stimulation of non- direction-specific motion reversals was introduced in the design of the spatial selective attention based steady-state BCI system. Here the “steady-state” brain responses were evoked by mirror movements which oscillated in two opposite directions. This stimulation protocol was also called the motion reversal and was temporally sinusoidally modulated by distinct frequencies in this paper. For the presentation of such oscillating motion, each of the two motion directions would be presented half cycle time and then be replaced by the other direction of motion, comprising one stimulus period. The direction change rate, which was specifically called the motion reversal frequency, served as the fundamental frequency of the motion reversals. Its first subharmonic frequency equaled the sinusoid frequency [12].

**Figure 1:**
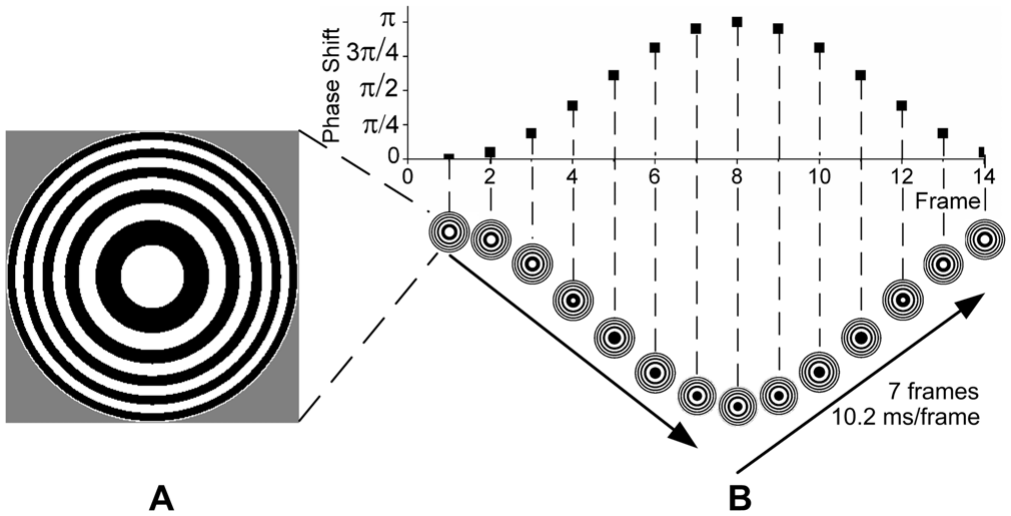
Motion reversal stimulation. (A) A Newton's ring based stimulator. (B) The motion reversal procedure of the rings. It was illustrated with a 14 Hz motion reversal frequency and each reversal contained 7 frames. The phase of the Newton's ring was modulated by a sinusoid of 7 Hz in [0, π] to produce motion reversals.

Compared with typical stimulation protocols of flickering and contrast changes that are used to evoke normal SSVEPs, many motion modes can be used to elicit SSMVEPs. In our research, the Newton's ring, which is an optical interference pattern widely existing in the natural world, was adopted as the template for motion reversal stimulation. It appears as a series of concentric and alternate bright and dark rings, where the outer rings are spaced more closely than inner ones. The phase of the Newton's ring was temporally sinusoidally shifted so as to produce the motion reversal procedure, which included the inward contraction and outward expansion motions alternately. Here the contraction of Newton's ring was implemented by its phase shift from 0 to π, and then expansion motion was achieved with phase shift from π back to 0. The position of the rings was not exactly repeated before and after a reversal and one displacement was inserted to maintain consistency of motion. Figure 1 showed a Newton's ring based stimulator and its motion reversal procedure in one stimulus period. Presentation of the stimulators and their reversals were controlled by the Psychophysics Toolbox 3.0 [13], [14].

### 3 Experimental Setups

Our BCI design utilized the Newton's ring to construct four visual stimulators and displayed them on a 21″ EIZO FlexScan T966 CRT monitor with a high refresh rate (setting 100 frames/s, measured ∼98 frames/s). The viewing distance was 70 cm. Each stimulator subtended a circular field of 4.8 degrees diameter and was composed of five symmetrical black and white rings. The stimulus luminance was 118 cd/m^2^ for the white rings, and 0.7 cd/m^2^ for the black ones (Michelson contrast of 98.8%). The squared values of the relative radius of all rings constituted an arithmetic progression and then each ring shared the same size except the most inner circle. When the stimulator moved, its mean luminance kept almost the same throughout the entire stimulus period but litter jitter would appear due to the area change of the inner circle. In the whole experiment, spatially homogeneous grey background with a luminance of 38 cd/m^2^ was displayed in pauses and around the stimulators.

Considering the hypothesis that steady-state responses can be explained by the temporal superposition of transient single-stimulus responses [15], [16], the destructive superposition of P1 and N2 components that were embedded in transient mVEPs elicited by individual motion reversals had great influence on the selection of motion reversal frequencies. For P1 latency of about 100 ms and N2 latency of 150–200 ms [5], [17], [18], a late N2 component evoked by former motion reversal may coincide with an early P1 component evoked by latter motion reversal when a specific motion reversal frequency was adopted and hence SSMVEPs disappeared. Further increase of motion reversal frequency would lead to destructive superposition of multiple N2 and P1 components that were evoked by a sequence of motion reversals and result in overall response decline within a wide frequency range [15]. Therefore, the inter-reversal interval of less than 50 ms would lead to decline or extinction of SSMVEPs to a great extent and the motion reversal frequency before 20 Hz was adopted in this paper.

**Figure 2:**
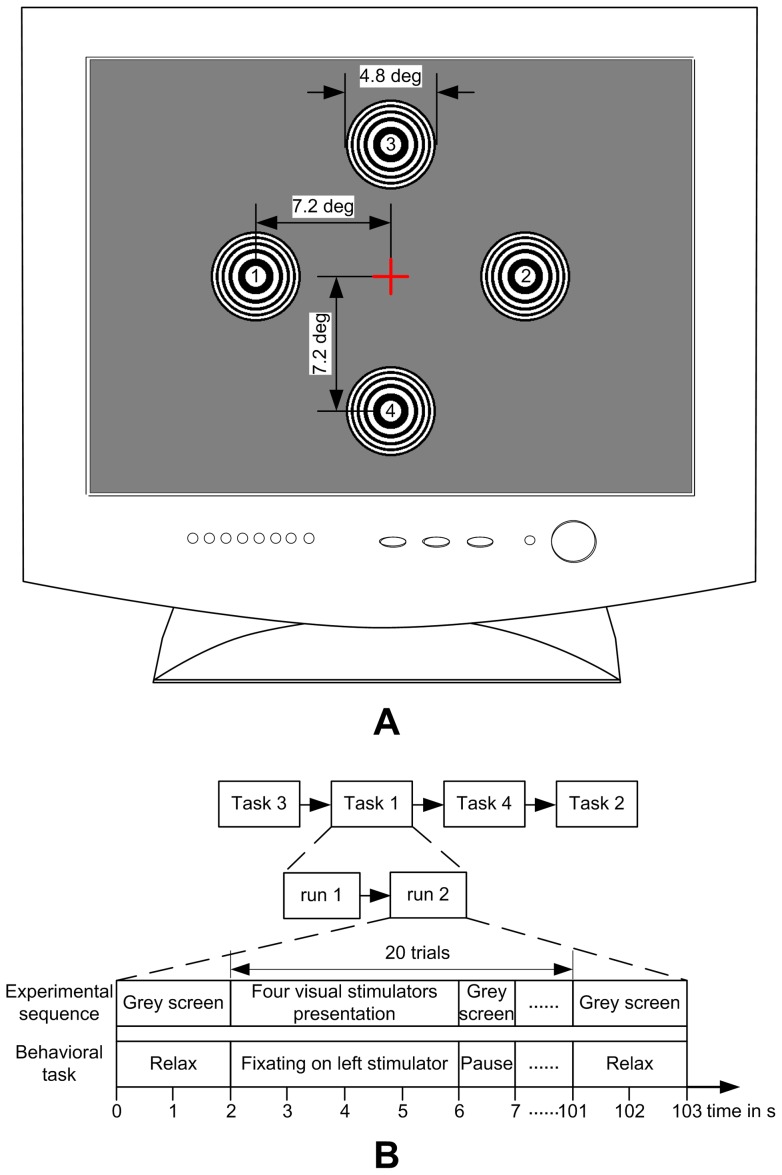
Schematic diagram of the experimental setup. (A) Distribution of four stimulators on the computer screen. The cross indicating the center of the monitor was not presented on the screen. (B) The timing of the experimental sequence and behavioral task. For each subject, four experimental tasks were imposed.

In this paper, four stimulators were uniformly spaced in left, right, up and down directions to the center of the monitor. The distance from the center of each stimulator to the center of the monitor was 7.2 degrees of visual angle (see Figure 2A). Four distinct motion reversal frequencies of 8.1, 9.8, 12.25 and 14 Hz, which were compatible to the measured refresh rate of 98 Hz, were presented as the fundamental frequencies of the four stimulators (left, right, up and down). Each frequency was obtained from dividing the refresh rate by the frame values in half cycle period (i.e. 12, 10, 8 and 7 frames), respectively.

### 4 Subjects and Recordings

Six right-handed healthy male subjects (age 23–29) participated in the experiments. They were requested to sit on a comfortable armchair in an ordinary lighting office room with no electromagnetic shielding. All of them had normal or corrected-to-normal eyesight and experienced BCIs before. As the traditional SSVEP-based BCIs, subjects in the experiments were asked to binocularly view the screen and fixate on the center of the target stimulator. They were also instructed not to track the stimulator movement with their eyes.

In previous mVEPs studies, the standard EEG positions of O1, Oz, O2, PO7 and PO8 were often used [5], [12]. In this study, one subject (subject S1) was chosen in advance to perform an eleven-electrode experiment to determine the activated areas that were mostly related to SSMVEP activities. The electrode array started on Oz site and then extended in steps of about 6 cm to the rest of the parietal-occipital region, which was also the area that the traditional SSVEPs originated from. Therefore, T7, C3, Cz, C4, T8, P3, Pz, P4, PO7, Oz and PO8 sites in International 10–10 System were adopted for this subject and the most activated sites in the experiment were later used for EEG recordings of other subjects. EEG signals were referenced to a unilateral earlobe, grounded at frontal position (Fpz), and sampled at 1200 Hz using a g.USBamp (g.tec Inc., Austria) system. They were also online band-pass filtered from 2 to 100 Hz and notch filtered between 48–52 Hz to remove artifacts and power line interference. All electrode impedances were kept below 5 kΩ during experiments.

For each subject, four experimental tasks were carried out where Task 1 to Task 4 was to focus attention on the visual stimulator of 8.1, 9.8, 12.25 and 14 Hz, respectively. Each task contained two runs and each run had twenty trials inside. Subjects were instructed to fixate on a specific stimulator throughout the task and Task 1, 2, 3 and 4 were performed one by one in random order (see Figure 2B). In the experiment of each run, 2 s of grey screen was first displayed, and then the four stimulators were simultaneously presented 4 s as a single trial. Two adjacent trials were isolated by grey screen and the interval time was fixed to 1 s. Before the run ended, another 2 s of grey screen was displayed. The total time of a single run would last 103 s and the whole experiment of each subject usually lasted about 20 min, depending on the inter-run interval governed by subjects. They were asked to blink or move their bodies only at that time. Therefore, the horizontal or vertical EOG signals were not necessarily recorded and trials contaminated by few artifacts were also not excluded.

### 5 Analyses

To investigate the applicability of the proposed SSMVEP-based BCI, we implemented Canonical Correlation Analysis (CCA) for offline target detection. CCA is a nonparametric multivariable method [19] to reveal the underlying correlation between two sets of multidimensional variables. It finds a pair of linear transforms for the two sets such that the transformed two sets have maximum correlation. Compared with traditional SSVEP recognition methods, it combines two steps of feature selection and frequency recognition, and therefore simplifies the experiment procedure with no need of subject-specific training [20]–[22]. Recently it has been widely used in multi-channel SSVEP-based BCIs.

In this paper, CCA was adopted to measure the correlations between stimulus frequencies and multi-channel EEG signals rather than to estimate the magnitude of a periodic component encoded in the brain signals [20]. This relied on the existence of the same response frequencies as the stimulus frequency and its harmonics. When one stimulator was attended, an enhanced correlation between its stimulus frequency and EEG signals was expected to occur, leading to a largest correlation coefficient among all stimulators. The stimulator with the largest coefficient was recognized as the target and the detection procedure was performed on each trial EEG data.

Suppose that there are 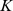
stimulus frequencies 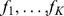 in the BCI, for the calculation of correlation coefficients between stimulus frequency 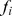
(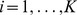) and EEG responses, two sets of signals are introduced into CCA. One set is the EEG signals 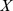 recorded from
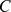
different channels with time window of 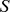 sample points, and the other set is the stimulus signals 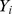, which are composed of sinusoids and cosinusoids pairs at the same frequency of the stimulus and its harmonics. Stimulus signals 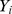 are constructed as
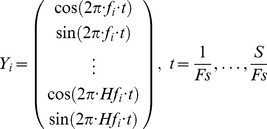
(1) where 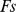 is the sampling rate, and 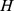 is the number of harmonics, which is dependent on how many frequency harmonics existed in SSMVEPs.

Given the multidimensional variables of 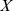 and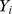, and their linear transformations 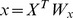 and 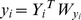, CCA can find the weight vectors 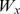 and 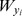 to maximize the canonical correlation of 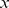
and 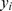 (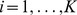) through solving the following problem
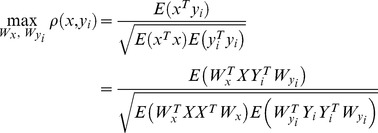
(2)


The maximum of 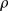, which corresponds to the maximum canonical correlation between 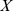 and 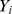, is taken as the recognition basis for stimulus frequency 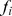 (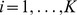).

When CCA is performed separately on each stimulus frequency 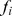 (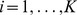) and the respective maximum correlation coefficient 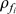 is obtained, the target with stimulus frequency of 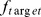 can be judged by
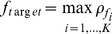
(3)


An important criterion to evaluate a BCI system is the ITR which measures the achievable information rate per unit time, given the detection accuracy and the time required making a decision. In this study, the offline ITR in bits/min [23] was adopted to evaluate the performance of the BCI system. It can be calculated as
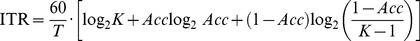
(4)where 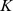 is the number of stimulators, 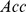 is the mean accuracy averaged over all stimulators and 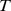 is the decision transfer interval (i.e. the sum of single detection time and interval between detections).

## Results

### 1 Spatio-Spectral-Temporal Characteristics of SSMVEPs

**Figure 3:**
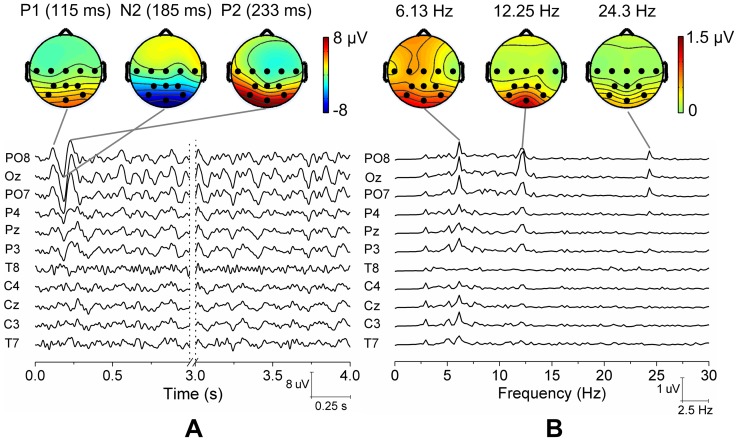
Time series and Topographies from eleven-channel mean SSMVEPs of subject S1. (A) Time series of the mean SSMVEPs. Topographic distributions exhibited symmetrical activations of the transient P1, N2 and P2 components. (B) Spectral topographies of three predominant frequency components of the mean SSMVEPs. The most activation area was also symmetrically located on PO7, Oz and PO8 sites.

Before extracting single-trial data from continuous EEG signals, the signals were first band-pass filtered between 3–30 Hz (4th order zero-phase-shift Butterworth filter) to minimize low frequency components. By averaging two-run datasets from eleven-channel recordings of subject S1, the time series and topographies of the mean SSMVEPs, which were elicited by 12.25-Hz motion reversals, were illustrated in Figure 3. The mean SSMVEPs in Figure 3A showed three strong transient motion-related components of P1, N2 and P2 with latencies of 115, 185 and 233 ms after onset of the motion reversal procedure, especially on PO7, Oz and PO8 sites. Corresponding topographic distributions [24] of these components exhibited symmetrical activations in the occipital lobe, which were caused by the isotropic radial (i.e. expansion and contraction) motions rather than hemispheric asymmetry caused by unidirectional translation motions, as anticipated by previous study [8]. Similar symmetrical activations of first subharmonic (6.13 Hz), motion reversal (12.25 Hz) and second harmonic (24.3 Hz) frequency components of the mean SSMVEPs, which maintained steady state 300 ms after motion reversal onset, can be observed in the spectral topographies [24] in Figure 3B. The three frequency components were predominant in the amplitude spectra and the responses at fundamental reversal frequency were the largest. The most activation sites of the three frequency components were also PO7, Oz and PO8.

### 2 BCI Performances

To avoid the introduction of less relevant EEG features into CCA, only electrode positions with strong steady-state responses were involved. The PO7, Oz and PO8 sites were selected for EEG recordings and CCA calculations of all subjects. In CCA calculations, the stimulus frequency 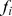
(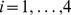) was selected as the motion reversal frequency of each stimulator, the channel count of 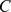 was selected as 3, and the harmonics of 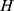 was selected as 0.5, 1 and 2. Under the time window of 4 s per trial, the offline detection accuracy of each run was estimated as the percentage of correctly judged trials. The procedure was repeated over all runs and thus eight accuracy values were obtained for each subject. The mean and standard deviation (SD) of the accuracy values of each subject was illustrated in Table 1, where subject S1 had the highest mean accuracy and subject S6 was the lowest. It showed that the detection accuracies of most subjects maintained high except subject S6 (*p = *0.0006, one-way analysis of variance (ANOVA)), and the mean accuracy and standard deviation over all subjects and all runs was 86.56±9.63%.

**Table 1:** Offline detection accuracies on fixed time window length (4 s).

Subjects	Accuracy (mean ± SD) (%)
S1	93.75±5.18
S2	93.13±7.04
S3	87.50±5.98
S4	85.63±10.50
S5	83.13±8.84
S6	76.25±8.76 *
Average	86.56±9.63

The asterisk (*) indicates statistically low accuracies of subject S6 as assessed by one-way ANOVA (*p* = 0.01).

The highest ITR of each subject was calculated under the trade-off between the time window length and corresponding mean accuracy averaged over eight runs. It was a theoretical offline value [9], [25] for which the time window was selected from the first second of the trial to the entire trial duration of 4 s in steps of 0.5 s. The interval time between time windows was adopted as the inter-trial interval of 1 s. For each time window length, the corresponding mean accuracy in each subject was calculated and then an individual ITR can be obtained, where the decision transfer interval 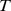 was set as the sum of the time window length and the interval time. Results in Table 2 demonstrated each subject's highest ITR under optimal time window length and mean accuracy, where all subjects shared approximately equivalent time window length to achieve comparable ITRs except subject S6. The ITR (mean ± SD) over all subjects was 15.93±3.83 bits/min.

**Table 2:** Offline highest ITRs on optimal time window length.

Subjects	Time window (s)	Accuracy (mean) (%)	ITR (bits/min)
S1	4	93.75	18.76
S2	3	90.00	20.59
S3	3	85.63	17.67
S4	4	85.63	14.14
S5	3	80.00	14.42
S6	4	76.25	9.99
Average	3.5±0.5	85.21±6.38	15.93±3.83

### 3 Response Significances and Inter- and Intra-Subject Variability

**Figure 4:**
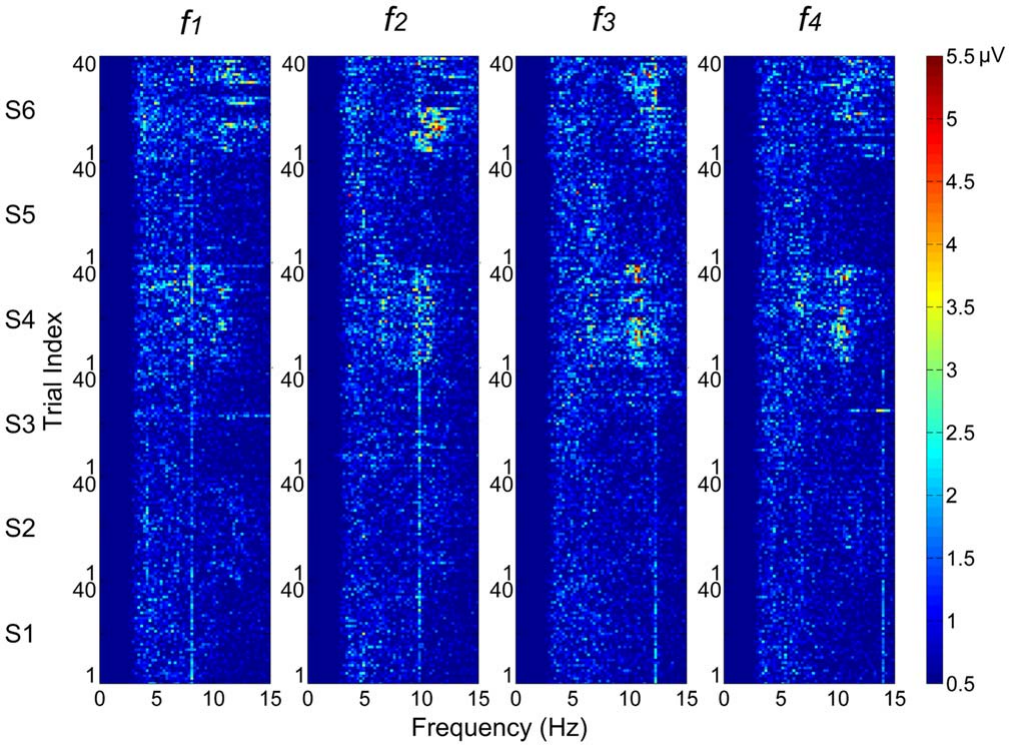
Image plot of amplitude spectra of SSMVEPs (*f*_1_ –***f*** **_4_) on Oz site.** Consecutive non-overlapped trials were sorted by ordinal run order from subject S1 to subject S6.

The statistical significance and variability of the SSMVEP responses is very critical for the applicability of BCI applications. Figure 4 presented the image plot of amplitude spectra of SSMVEPs (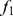
–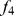) in frequency range of 3–15 Hz, which were calculated by Fast Fourier Transform (FFT) of consecutive non-overlapped trials from Oz site. FFTs revealed that responses at motion reversal frequencies were predominant in the amplitude spectra. For most subjects, the relatively consistent motion reversal frequency related responses can be observed across trials, but some responses were also not appreciable from fluctuation noise. On the statistics of responses at motion reversal frequencies, a rigorous statistical criterion of Circular Hotelling's T-Squared test (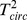) [26] was used to assess the amplitude and phase consistency of stimulus-locked SSMVEPs that differed from noise, and to determine significance of differences between stimulus-locked and non- stimulus-locked responses. The run-to-run response variability within a subject was also assessed by 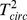. For the response at each particular motion reversal frequency, as its Fourier coefficient computed from each trial was a pair of real and imaginary numbers corresponding to the magnitude of the amplitude and the orientation of the phase, two-run estimates of such complex Fourier coefficients were converted to bivariate vectors and then submitted to 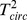 to assess how the amplitude and phase variations under different conditions could be. If the response vectors are sufficiently large with coherent phases so as to exceed the 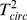 criterion at a significance level of 0.01, steady-state responses are evaluated significantly strong from zero. In this paper, most subjects' stimulus-locked responses exhibited statistically consistent amplitudes and phases over each of the four motion reversal frequencies (20 of 24), except subject S6 at 8.1 Hz (*p* = 0.751), subject S5 and S6 at 9.8 Hz (*p* = 0.331 and *p* = 0.073), and subject S6 at 14 Hz (*p* = 0.075). When comparing the responses at stimulus frequency with the responses at other frequencies, or estimating the run-to-run response variability within a subject, the statistical significance of difference between responses in two conditions would also be assessed by 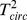 at the significance level of 0.01. For most subjects, the stimulus-locked responses were significantly larger than the responses at frequencies of non-target stimuli (19 of 24), except subject S6 at 8.1 Hz (target vs. non-targets: *p* = 0.817), subject S5 and S6 at 9.8 Hz (target vs. non-targets: *p* = 0.837 and *p* = 0.022), and subject S4 and S6 at 14 Hz (target vs. non-targets: *p* = 0.030 and *p* = 0.071). We also found that no significant intra-subject response variability between two individual runs of each subject existed (22 of 24), except subject S4 at 8.1 Hz (*p*<1.0×10^−5^) and subject S6 at 9.8 Hz (*p*<1.0×10^−3^). The inter-subject variability, which was assessed by one-way ANOVA and Tukey HSD post-hoc comparisons, revealed that there were significant differences between amplitude data of individual subjects over each of the four motion reversal frequencies (*p*<1.0×10^−5^ at 8.1 Hz, *p*<1.0×10^−11^ at 9.8 Hz, *p*<1.0×10^−10^ at 12.25 Hz and *p*<1.0×10^−19^ at 14 Hz). It indicated the variability between subjects was much larger than the variability within a subject.

### 4 Adaptation Effect

It is well known in neurophysiology that a repetitive stimulation tends to evoke much weaker responses after susceptible adaptation. It is an important issue in the design of SSVEP-based BCIs where a prolonged inspection of a repeated stimulation is needed. In physiological studies, adaptation responses consist of an initial increase followed by a decline and there exists several seconds delay before the initial peak is reached [10], [11], [27]. The declining adaptation process lasts a wide range of time scales from tens of milliseconds to minutes, depending on the stimulus property [27], [28]. More prolonged adaptation results in stronger response decline. For the steady-state motion reversal stimulation, Heinrich and Bach indicated that it can overcome the high susceptibility to adaptation that existed in direction-specific transient motion stimulation [12]. In this paper, a quantitative investigation of to what extent the amplitudes of SSMVEPs were affected by adaptation was made. Because the differences of SSMVEP amplitudes between subjects were larger than that within a subject, adaptation analysis was implemented on the time course of the mean amplitudes for each motion reversal frequency and each subject separately, where the mean amplitudes were computed by averaging two-run amplitude data of the same trial order. The averaged time course with 5-s interval revealed how the amplitudes evolved over a 100-s stimulation sequence. We fitted the transition with single exponential to approximate the adaptation process and the decline from initial peak amplitude to subsequent sustained portion was characterized by the time constant. The exponential fitting was constructed as according to [28].
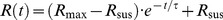
(5)where 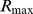 was taken as the initial peak value of the time course and corresponded to the starting point of declining exponential fitting, 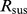 was the sustained portion of the fitted exponential and
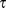
was the time constant.

In order to find the point that the declining adaptation process initially started from, first an asymmetric Laplace distribution function, which is expressed as splicing of increase and decline exponentials back-to-back, was used to fit all of the 100-s time courses (least-squares fit). The results of all fittings demonstrated that each splicing position, which indicated where the time course started to decline, belonged among the first three data points (i.e. the time range of 0–10 s). Therefore, the local peak point near the splicing position, which was also in the range of the first three data points, was taken as the starting point to fit the declining adaptation process.

We quantified the adaptation effect with the initial-to-sustained ratio (ISR) [28].
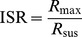
(6)


**Figure 5:**
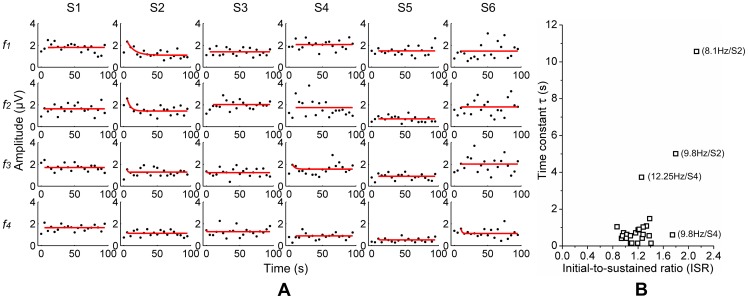
Adaptation fittings of mean time courses and the corresponding ISR/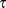
plot. (A) The mean time courses and fitted exponentials of SSMVEP amplitudes on Oz site. Each symbol represents the mean amplitude averaged in the same trial order. (B) Scatter plot of the relationship between ISR and time constant
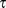
for each motion reversal frequency and each subject. Each symbol represents an ISR/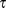
pair.

For each motion reversal frequency and each subject, the mean time courses of SSMVEP amplitudes and their least-squares fitting of declining exponentials were displayed in Figure 5. According to equation 5, an ISR value of near 1.0 with a
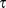
value of near 0 implies small or no difference between initial peak amplitude and sustained portion of fitted exponential, while exponential would approximately degenerate into horizontal line. It was the signature of low adaptation effect. Condition far from these values indicated the occurrence of adaptation and its level can be measured by the deviation. In Figure 5A, the comparisons between different time courses revealed that the majority of time courses exhibited shallow curves with unconspicuous initial peaks, to which horizontal lines were fitted. Their ISR/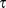
pairs in Figure 5B were closely grouped around an area with ISR value ranging from 0.87 to 1.41 (mean ± SD: 1.16±0.16) and
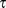
value ranging from 0.15 to 1.48 s (mean ± SD: 0.63±0.36 s). This meant no distinct adaptations occurred (20 of 24). Large exceptions were found on subject S2 at 8.1 Hz (ISR/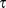: 2.13/10.56) and 9.8 Hz (ISR/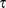: 1.80/5.01), where they exhibited considerable deviations from the group in both coordinates. This meant remarkable adaptations of SSMVEPs, which corresponded to large exponential fittings of subject S2 at 8.1 Hz and 9.8 Hz in Figure 5A. Intermediate adaptation effects can only be found on subject S4 at 9.8 Hz (ISR/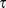: 1.75/0.60) and 12.25 Hz (ISR/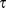: 1.26/3.73), where they solely exhibited either a high ISR or a large
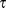
from the group. These findings revealed none or weak adaptations existed in most conditions.

## Discussion

Our study, for the first time, assessed the applicability of implementing SSMVEP as a promising neurophysiologic signal for BCI applications. SSVEP elicited by flickering light in low-frequency range (<15 Hz) has high signal-to-noise ratio (SNR) [29], a wide distribution over the scalp, and it can be evoked easily. However, it also brings larger perceived brightness [30]. That is called the Brücke-Bartley effect that flickering light of approximately 10 Hz is perceived brighter even than steady light of the same luminance. It would make subjects uncomfortable and cause visual fatigue. The motion reversal paradigm, which utilized position changes rather than luminance alternations, resembled the pattern reversal paradigm with constant mean luminance throughout stimulation. But compared with the sudden phase inversion of checkerboard pattern reversal, the phase of Newton's ring shifted gradually, which meant lower stimulus frequency resulted in slower position changes. The slow position changes in low-frequency range made less discomfort to subjects, while good SNR can be simultaneously maintained. The use of isotropic radial motion of circular stimulator not only facilitated central fixation of subject's attention to reduce optokinetic nystagmus caused by involuntary tracking of lateral motion [31], but also may stimulate additional cortical (e.g. the vestibular) areas to produce stronger SSMVEP responses [32]. Due to radial motion, the symmetrical activation of SSMVEP responses among PO7, Oz and PO8 sites can be observed across each motion reversal frequency and each subject, and the responses on Oz site were the largest. It facilitated the design and application of SSMVEP-based BCIs that electrodes can simply be arranged on the midline of the scalp.

The results of response recognition and statistical evaluation demonstrated that the utilization of oscillating Newton's rings provided a fast way of implementing motion-related BCIs with significantly strong responses and without suffering from adaptations. Due to oscillations of motion in opposite directions, motion reversals can effectively overcome the high susceptibility to direction-specific adaptation caused by unidirectional motion. The fairly long recovery interval between trials was no longer needed. The finding of overall low-adaptation characteristics in this paper can be a featured advantage to reduce the complexity of sophisticated motion-related BCI designs. With the utilization of CCA, the SSMVEP detection procedure took short time (3–4 s) to efficiently recognize the target on single trial level and all subjects except subject S6 exceeded the level of 80% mean accuracy, which were comparable to the performances of conventional four-choice SSVEP-based BCIs [22], [25]. Similar to CCA results, response quantification and statistical evaluation in frequency domain also revealed significantly strong amplitude and phase consistency throughout most motion reversal frequencies in subject S1–S5, while subject S6 lacked significant responses at most stimulation frequencies (i.e. 8.1, 9.8 and 14 Hz). The absence of strong evoked neural activity in this subject may lead to such lack of significant responses and the lower recognition accuracies. For subject S4 and S5, the lack of significant responses only emerged on a single stimulation frequency. As previous illustration on P1 latency of about 100 ms and N2 latency of 150–200 ms, inter-reversal interval between 50 and 100 ms would probably lead to fully destructive superposition of single N2 and P1 components and cause SSMVEPs extinction on a specific stimulation frequency between 10 and 20 Hz, which depended on the specific P1 and N2 latency in each subject. In this paper, the lack of significant responses at 14 Hz in subject S4 and 9.8 Hz in subject S5 may be caused by such fully destructive superposition, where the specific N2-P1 latency difference of subject S4 accorded with inter-reversal interval of 14 Hz and N2-P1 latency difference of subject S5 with inter-reversal interval of 9.8 Hz. This implied that motion reversal stimuli presented in this paper could be able to evoke significantly strong oscillatory activities for BCI applications and the absence of steady-state responses at a specific stimulation frequency in certain subject may simply attribute to the selected stimulation frequency that brought destructive superposition of individual mVEPs. Although statistical significant test also indicated large response differences among different subjects, the CCA method was less influenced and the comparable accuracy results among subject S1–S5 may benefit from the combination usage of multiple harmonic frequencies. The relatively low offline ITRs in this paper were mainly derived from the finite target number and an adequate inter-trial interval. A shorter (<0.2 s) inter-trial interval was proven to be sufficient for the online CCA calculation [20] and can be adopted to promote the ITRs; but its influence on the adaptation effect needs further study.

In summary, we proposed a novel VEP-BCI paradigm based on steady-state motion reversals, which had different neurophysiological background but the features of comparable performance, low-adaptation characteristics, less visual discomfort and significantly strong responses for single-trial detection, as compared with traditional SSVEP-based BCIs. Further studies will focus on the development of an online version with enhanced reliability and efficiency.
